# Dysregulated NK cell activation and myeloid-lymphoid imbalance underpin COPD progression: insights from high-dimensional immune profiling and smoking-induced immune remodeling

**DOI:** 10.3389/fimmu.2025.1623319

**Published:** 2025-09-19

**Authors:** Qin Qiao, Yazheng Yang, Ke Huang, Hongtao Niu, Xiaoxia Ren, Shiwei Qumu, Wei Li, Chen Dong, Ting Yang, Ling Ni

**Affiliations:** ^1^ Institute for Immunology and School of Basic Medicine, Tsinghua University, Beijing, China; ^2^ National Center for Respiratory Medicine, State Key Laboratory of Respiratory Health and Multimorbidity & National Clinical Research Center for Respiratory Diseases, Beijing, China; ^3^ Institute of Respiratory Medicine, Chinese Academy of Medical Sciences, Beijing, China; ^4^ Department of Pulmonary and Critical Care Medicine, Center of Respiratory Medicine, China-Japan Friendship Hospital, Beijing, China; ^5^ Westlake University School of Medicine-affiliated Hangzhou No. 1 People’s Hospital, Hangzhou, Zhejiang, China

**Keywords:** peripheral blood, flow cytometry, RNA-seq, smoking, immune perturbation

## Abstract

**Background:**

Chronic obstructive pulmonary disease (COPD) is a significant global health concern, marked by persistent inflammation and immune dysregulation. Although it is widespread and has substantial clinical implications, the systemic immune mechanisms driving disease progression are not fully understood. Since blood contains a diverse array of immune cells and offers a non-invasive means of assessing immune homeostasis and overall physiological status, investigating immune dysregulation through blood sampling offers considerable value for both basic research and clinical application. This approach can provide novel insights into the pathogenesis of COPD.

**Methods:**

This study employed high-dimensional flow cytometry and RNA sequencing to comprehensively characterize peripheral immune cells from a cohort of 69 COPD patients spanning clinical stages 1 to 4, alongside 41 healthy donors as controls. To capture granulocyte populations typically excluded from peripheral blood mononuclear cell analyses, fresh whole blood samples were analyzed directly.

**Results:**

Our study revealed a marked shift in the myeloid-lymphoid balance, characterized by elevated neutrophils, eosinophils, and classical monocytes that correlated with disease severity, alongside reduced CD8^+^ T cells and circulating T follicular helper cells. Transcriptional profiling identified oxidative stress pathways, T cell suppression, and aberrant natural killer (NK) cell activation as hallmarks of advanced COPD. Notably, activated Nkp44^+^ NK cells were significantly enriched in severe stages, implicating their role in perpetuating inflammation. Smoking exacerbated immune perturbations, including upregulated complement activation and B cell pathways, though cessation partially restored transcriptional homeostasis.

**Conclusions:**

This study underscores the value of peripheral immune profiling in capturing the heterogeneity of COPD. The results reveal systemic immune dysregulation-especially NK cell hyperactivity and point to potential therapeutic avenues aimed at modulating immune responses to slow disease progression.

## Introduction

Chronic obstructive pulmonary disease (COPD) is a chronic airway inflammatory disease that leads to lung tissue damage and airflow obstruction (1). It is predominantly caused by prolonged exposure to irritating gases or particulate matter, with cigarette smoking being the most significant risk factor (2). According to the World Health Organization estimates published in 2024, COPD is the fourth leading cause of death worldwide in 2021 (3). Furthermore, a study published in 2023 projected that the prevalence of COPD among individuals aged 25 years and older would increase by 23% from 2020 to 2050 (4).

COPD is a complex and heterogeneous disease, distinguished by a spectrum of pathological mechanisms and diverse clinical presentations. Immunological pathways have been increasingly recognized as integral to the pathogenesis of COPD. The continuous and progressive activation of both innate and adaptive immune responses perpetuates the chronic inflammation observed in the airways and lung parenchyma (5–8). Early studies primarily focused on innate immune cells including neutrophils, macrophages, dendritic cells, natural killer (NK) cells, and eosinophils in the pulmonary environment (9). Subsequent studies have unveiled correlations between COPD and elevated levels of CD8^+^ and CD4^+^ T cells, as well as B cells, in the airways and lung tissue (5, 10, 11). In recent years, COPD has garnered attention as a disease associated with chronic systemic inflammation (6, 12, 13). Therefore, it is imperative to investigate the immune cells in the peripheral circulation during the progression of COPD, which will facilitate the elucidation of the intricate interplay between pulmonary and systemic immune responses that is characteristic of the disease.

Previous studies have investigated changes in peripheral blood mononuclear cells (PBMCs) in the context of COPD (11, 14). However, these studies, which focused primarily on PBMCs, often failed to include granulocyte populations. Additionally, these studies often had relatively small sample sizes and lacked a comprehensive set of clinical benchmarks. Given these limitations, a more comprehensive understanding of the pathogenesis of COPD necessitates conducting in-depth immunophenotyping of whole blood samples, thereby providing a holistic view of immune cell dynamics in COPD.

In this study, we utilized high-dimensional flow cytometry to comprehensively visualize the myeloid and lymphoid immune cell compartments in a cohort of 69 COPD patients presenting with a wide range of clinical manifestations, from mild to severe disease stages. This in-depth immunophenotyping revealed significant alterations in the proportion and functional status of key immune cell populations, including neutrophils, eosinophils, classical monocytes, CD8^+^ T cells, T follicular helper (Tfh) cells, and NK cells, across the spectrum of disease severity. Additionally, supplementary bulk RNA-seq analysis further elucidated the impact of oxidative stress, T cell perturbation, and aberrant activation of NK cells on disease progression.

## Materials and methods

### Subjects and classification

Sixty-nine patients diagnosed with COPD by a physician were recruited in the study. Their clinical information including age, sex, body mass index (BMI), smoking history, complication and pulmonary function test at the time of sampling were retrieved from patient clinical records (**Table 1**, **Supplementary Table S1**). In addition, blood samples from 41 healthy donors (HDs) were used as the healthy control.

**Table 1:** Characteristics of the COPD patients recruited in this study.

Characteristics	COPD (GOLD stage)		
	1	2	3-4
Number of patients	19	26	24
Current smokers	5	6	9
Quitters	9	14	12
Never smokers	4	5	3
Not available	1	1	0
Age (years)^†^	64.58 ± 2.29	67.04 ± 2.08	62.88 ± 1.80
Number of males	12	19	21
BMI (kg/m^2^)^†^	24.89 ± 1.11	25.70 ± 0.80	22.76 ± 1.05
Smoking (Pack-year smoked)^†^	27.05 ± 6.25	29.98 ± 5.59	34.15 ± 6.00
FEV_1_/FVC (%)^†^	66.26 ± 1.25	53.03 ± 1.96	37.21 ± 2.78
FEV_1_ (% of predicted)^†^	87.71 ± 1.90	66.12 ± 1.74	35.43 ± 2.48

COPD, chronic obstructive pulmonary disease.

GOLD, the Global Initiative for Chronic Obstructive Lung Disease.

Quitters, those with a history of smoking for several years but who had quit for more than one year.

FEV_1_, forced expiratory volume in the first second; FVC, forced vital capacity.

^†^Values are presented as mean ± SE.

The airflow obstruction of patients was assessed using the ratio of the forced expiratory volume in the first second to the forced vital capacity (FEV_1_/FVC) after bronchodilator administration (15). FEV_1_ values are shown as a percentage of their expected values for age, gender, and height. Patients were classified into three distinct clinical severity groups according to the Global Initiative for Chronic Obstructive Lung Disease (GOLD) staging system: Stage 1 mild, FEV_1_ ≥ 80% of predicted; Stage 2 moderate, FEV_1_ = 50-79% of predicted; Stage 3-4: severe, FEV_1_ < 50% of predicted (15). The FEV_1_/FVC ratio should be <0.70 for all stages (15).

### Flow cytometry

Peripheral blood samples were collected from 69 patients diagnosed with COPD and 41 HDs using VACUETTE EDTA-coated blood collection tubes. For each flow cytometry panel analysis, a volume of 100 μL of whole blood was initially washed with phosphate-buffered saline (PBS) and treated with Fc blocking reagent. Cells were stained with a mixture of antibodies for 30 min in the dark at 4 °C. After incubation, 1.5 mL of 1 × BD FACS Lyse solution was added, mixed, and incubated for 10 min in the dark at room temperature. The samples were then centrifuged at 500g for 5 min; the supernatant was carefully aspirated. The cells were washed twice with 1.5 mL of PBS and centrifuged at 500g for 5 min each time. The prepared samples were transferred to FACS tubes and analyzed by a BD LSRII flow cytometer with 5 lasers.

The following fluorescence-conjugated anti-human antibodies were used for the three flow cytometry panels, respectively. Panel 1: anti-Lin1 CD3 (UCHT1), CD14 (HCD14), CD19 (HIB19), CD20 (2H7), CD56 (HCD56) FITC, anti-HLA-DR (L243) AF700, anti-CD11c (3.9) BV605, anti-CD123 (7G3) PE-CF594, anti-CD14 (MɸP9) APC-Cy7, anti-Siglec8 (7C9) Percpcy5.5 and anti-CD16 (3G8) PE-Cy5. Panel 2: anti-Lin2 CD3 (UCHT1), CD14 (HCD14), CD19 (HIB19), CD20 (2H7) FITC, anti-CD45 (HI30) Percpcy5.5, anti-CD56 (HCD56) PE-Cy5, anti-CD3 (UCHT1) AF700, anti-CD127 (eBioRDR5) PE-Cy7, anti-CD4 (RPA-T4) APC-Cy7, anti-CD294 (BM16) PE-CF594, anti-Nkp44 (p44-8.1) PE, anti-CXCR5 (RF8B2) BV421, anti-PD1 (EH12.1) BV605. Panel 3: anti-CD3 (UCHT1) AF700, anti-CD45 (HI30) PE-Cy7, anti-CD20 (2H7) BV605, anti-CD27 (0323) Percpcy5.5, anti-CD38 (HIT2) PE-Cy5, anti-CD43 (CD43-10G7) APC, anti-IgD (IA6-2) PE-CF594, anti-IgM (G20-127) BV421, Aqua live/dead viability dye was added to exclude dead cells.

Analysis was performed using FlowJo version 10.6.1. For Uniform Manifold Approximation and Projection (UMAP) (16) visualization, FCS files were firstly cleaned by FlowJo plugins [FlowClean (17) or FlowAI (18)], and further cleaned by manual gating to remove debris and dead cells. In each staining panel, an equal number of cells from each FCS file were extracted using DownSample plugin and then concatenated into one FCS file. UMAP analysis (16) was applied to this FCS file to generate a visualization map, which was subsequently used in the FlowSOM (19) clustering algorithm to identify each cell subset.

### RNA sequencing

After flow-cytometric analysis, we randomly selected 25 blood samples from those with sufficient remaining volume for downstream RNA-seq. These samples were derived from COPD patients classified as stage 1 (mild, n = 6), stage 2 (moderate, n = 12), or stages 3–4 (severe, n = 7) and were explicitly noted in **Supplementary Table S1**. Total RNA was extracted by TRIzol Reagent. The integrity of RNA was assessed using an Agilent 2100 Bioanalyzer and RNA purity was assessed using a NanoDrop spectrophotometer. The RNA samples were sent to BGI genomics for library construction (DNBSEQ Eukaryotic mRNA library) and sequencing (DNBseq), with a read length of 150 bp for paired-end reads. Read quality was verified using FastQC. Clean reads were aligned to the human GRCh38 reference genome using Hisat2, and uniquely mapped reads were summarized by feature Counts from the Subread package. Differentially expressed genes (DEG) were identified with edgeR and limma packages in R version 3.6.3, with criteria of at least log_2_(FC) > 1 and (false discovery rate) FDR < 0.05. Gene ontology analysis and pathway enrichment were conducted by Metascape online analysis platform (20) and https://www.bioinformatics.com.cn (last accessed on 10 Dec 2024), an online platform for data analysis and visualization (21).

### Statistical analysis

Data analysis and graphical representation were performed using Prism 9.0.0 software (GraphPad Software, La Jolla, CA). For statistical comparisons, Ordinary one-way ANOVA with Tukey’s test was used for parametric data, while Kruskal-Wallis with Dunn’s test was applied for nonparametric data. Pearson correlation coefficients were calculated to assess correlations between paired values. *P*-values < 0.05 are considered statistically significant.

The software used in this study, including FlowJo 10.6.1 software, Prism 9.0.0 software, and R 3.6.3 have been used with the appropriate licenses and permissions.

## Results

### Disease-related cell-composition changes in peripheral immune cells

**Figure 1:**
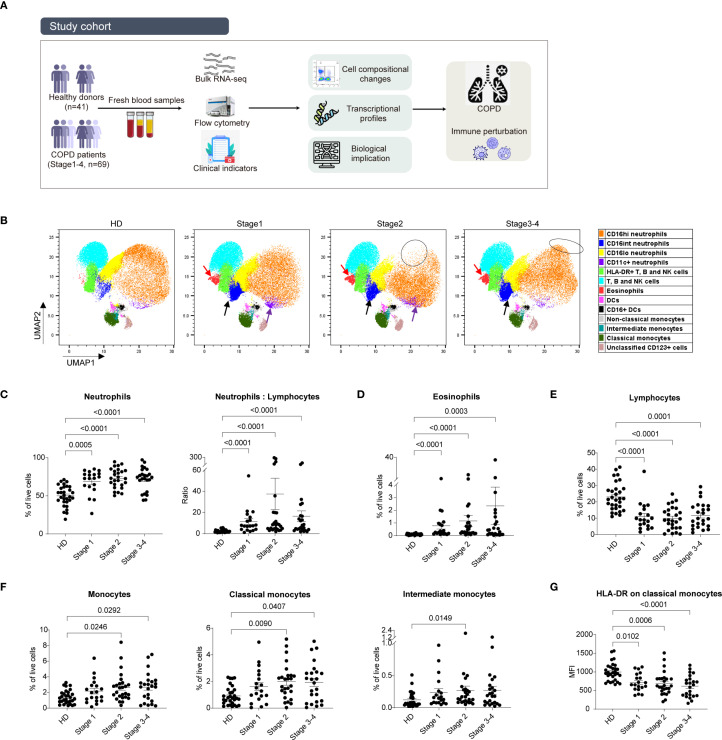
Disease-related cell-composition changes in whole blood from different stages of COPD patients and HDs. **(A)** Schematic diagram of overview of the study design. **(B)** UMAP analysis of flow panel 1 showed an overall view of immune cell subsets comparing different stages of COPD patients and HDs. UMAP projection of live cells from 15 HDs and 45 COPD patients (n = 15 for each group). **(C)** Percentage of neutrophils within live cells (left) and the ratio of neutrophils to lymphocytes (right). **(D)** Percentage of eosinophils within live cells. **(E)** Percentage of total lymphocytes within live cells. **(F)** Percentage of monocytes, classical monocytes, intermediate monocytes within live cells. **(G)** MFI of HLA-DR on classical monocytes. Scatter dot plots were presented with mean ± SE, the data of 30 HDs and 69 COPD patients (stage 1 n = 19, stage 2 n = 26, stage 3–4 n = 24) were included. Statistical analysis of the comparisons in **(C–G)** were performed using the non-parametric Kruskal-Wallis with Dunn’s multiple comparisons test by GraphPad (version 9.0.0). *P*-values were denoted and those lower than 0.05 are considered statistically significant.

To elucidate the immune perturbations associated with the progression of COPD and identify potential therapeutic targets, we enrolled 41 healthy donors and 69 patients with COPD spanning a spectrum of clinical severities, including 19 patients with mild COPD (stage 1), 26 with moderate COPD (stage 2), and 24 with severe COPD (stages 3-4). Patients’ clinical information is shown in **Table 1** and **Supplementary Table S1**. Blood samples were analyzed using multicolor flow cytometry and bulk RNA-seq (**Figure 1A**).

To profile cellular shifts across different clinical severities, we analyzed the whole blood samples with three staining panels designed to characterize myeloid and lymphoid cell subsets (**Supplementary Figures S1A–C**). Data was processed using an unbiased auto analysis function of FlowJo software which performed an UMAP analysis to visualize distinct immune cell subsets based on mean fluorescence intensity (MFI) of each marker within the defined clusters of cell subsets (**Supplementary Figures S2A–C**). UMAP and FlowSOM clustering of Panel 1 provided a comprehensive overview of the distribution of immune cell subsets across different stages of COPD patients and HDs (**Figure 1B**). This analysis revealed a marked divergence in the distribution of CD16^hi^ neutrophils in stages 2 and 3-4 (indicated by black circles), increased CD16^int^ neutrophils across all COPD stages (indicated by black arrows), and higher CD11c^+^ neutrophils in stage 1 and stage 2 (highlighted by purple arrows). Additionally, it also indicated a substantial increase in eosinophil count in all COPD stages (indicated by red arrows).

To corroborate these findings, a manual gating approach was employed to analyze neutrophils and eosinophils (**Supplementary Figure S1A**). Consistently, the percentages of neutrophils and eosinophils were augmented across all patient groups (**Figures 1C, D**). The neutrophil-to-lymphocyte ratio (22), a biomarker for COPD exacerbation, exhibited an elevation in all patient groups relative to HDs (**Figure 1C**). – Conversely, the proportion of total lymphocytes (Lin1^+^CD14^-^) significantly decreased across all disease groups compared with HDs (**Figure 1E**).

Similar to the findings in neutrophil populations, the proportion of total monocytes increased in two patient groups, paralleling the progression of disease severity (**Figure 1F**). We classified monocytes into classical, intermediate, and non-classical subsets based on the expression of CD16 and CD14 (23). Elevated frequencies of both classical (CD14^+^ CD16^-^) and intermediate (CD14^+^ CD16^int^) subsets were observed in patient groups compared to HDs (**Figure 1F**). To further character the antigen-presenting capacity and activation status of these monocyte subsets, we evaluated the expression level of HLA-DR. Notably, only the MFI of HLA-DR on classical monocytes was notably diminished across all disease stages, consistent with the progression of disease severity (**Figure 1G**).

Collectively, these findings suggest a correlation between augmented myeloid cell populations and concurrently diminished lymphoid immune cell counts, indicating a dysregulation of the homeostatic balance between myeloid and lymphoid lineages. Such a perturbation is hypothesized to contribute to the pathogenesis of uncontrolled, persistent chronic inflammatory responses.

### Distinct transcriptional profiles underscore the impact of oxidative stress and T cell perturbation in COPD pathogenesis

**Figure 2:**
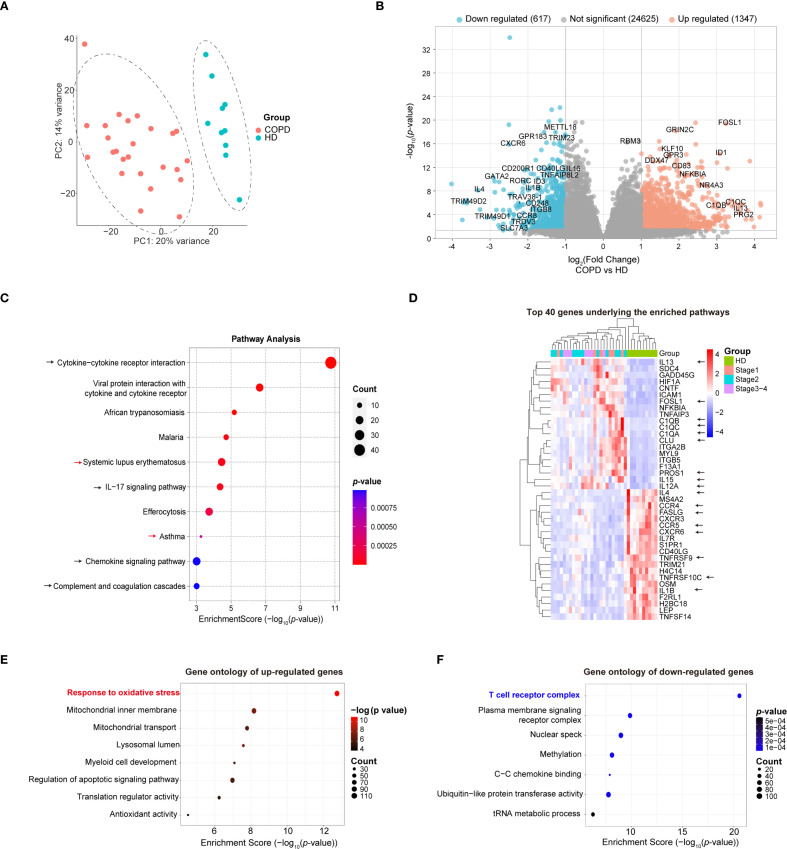
Bulk RNA-seq analysis of peripheral blood from HDs and COPD patients with stage 1-4. **(A)** PCA analysis to show the global variability of gene expression in each individual. (HDs, n = 10; stage 1, n = 6; stage 2, n = 12; stage 3-4, n = 7). **(B)** Volcano plot to show total 1964 DEGs between patients of stage1–4 and HDs. (log_2_(FC) < -1 and FDR < 0.05 were indicated by green, and (log_2_(FC) > 1 and FDR < 0.05 were indicated by red). **(C)** Pathway enrichment analysis to show the enriched pathways of the total DEG between patients of stage1–4 and HDs. (Enrichment Score > 3). **(D)** Heatmap to show the gene expression level of the top 40 genes with the most significance underlying the enriched pathways. **(E)** Gene ontology analysis to show the enriched GO terms of up-regulated genes. **(F)** Gene ontology analysis to show the enriched GO terms of down-regulated genes.

To elucidate the transcriptomic changes underlying COPD pathogenesis, we performed principal component analysis (PCA) to assess global gene expression variability among groups, which revealed a clear distinction between the disease groups and HDs (**Figure 2A**). Subsequently, differential gene expression analysis identified 1964 DEGs, with 1347 up-regulated and 617 down-regulated, based on a threshold of log_2_(FC) > 1 and FDR < 0.05 (**Figure 2B**, **Supplementary Table S2**). The functional analysis of these DEGs unveiled their involvement in a multitude of immune-related pathways, including cytokine-cytokine receptor interaction (e.g., *FASLG*, *TNFRSF9*, *TNFRSF10C*, *IL7R*, *IL4*, *IL12A*, *IL15*), IL-17 signaling pathway (e.g., *NFKBIA*, *TNFAIP3*, *FOSL1*, *IL1B*, *IL13*), chemokine signaling pathway (e.g., *CXCR3*, *CCR4*, *CCR5*) and complement and coagulation cascades (e.g., *F13A1*, *C1QA*, *C1QB*, *C1QC*, *CLU*, *PROS1*) (**Figures 2C, D**, **Supplementary Table S3**). More intriguingly, these DEGs were also enriched in autoimmune disease pathways, revealing a striking similarity between COPD pathogenesis and immune dysregulation in certain autoimmune diseases such as systemic lupus erythematosus and asthma (**Figure 2C**). This similarity underscores a strong correlation between the pathogenesis of COPD and the dysregulation of inflammatory responses and immune system homeostasis.

To further refine our understanding of the transcriptional changes, we performed separate gene ontology analyses on the up-regulated (**Figure 2E**) and down-regulated DEGs (**Figure 2F**). The analysis revealed a pronounced activation of pathways associated with the response to oxidative stress, thereby reaffirming the critical role of oxidative stress and oxidative damage in the pathogenesis of COPD (**Figure 2E**, **Supplementary Table S3**). Moreover, a notable downregulation of genes associated with the T cell receptor complex was observed, suggesting a potential reduction in T cell numbers or their functional efficacy as a significant factor influencing the pathological trajectory of COPD (**Figure 2F**, **Supplementary Table S3**).

### COPD-related alterations in T cell and natural killer cell subsets

Given the alterations in transcriptional profiles that elucidate the correlation between T cell suppression and the pathological progression of COPD, we performed flow cytometry to analyze T cell and NK cell subsets, which, similar to CD8^+^ T cells, exhibit cytotoxic effector functions.

**Figure 3:**
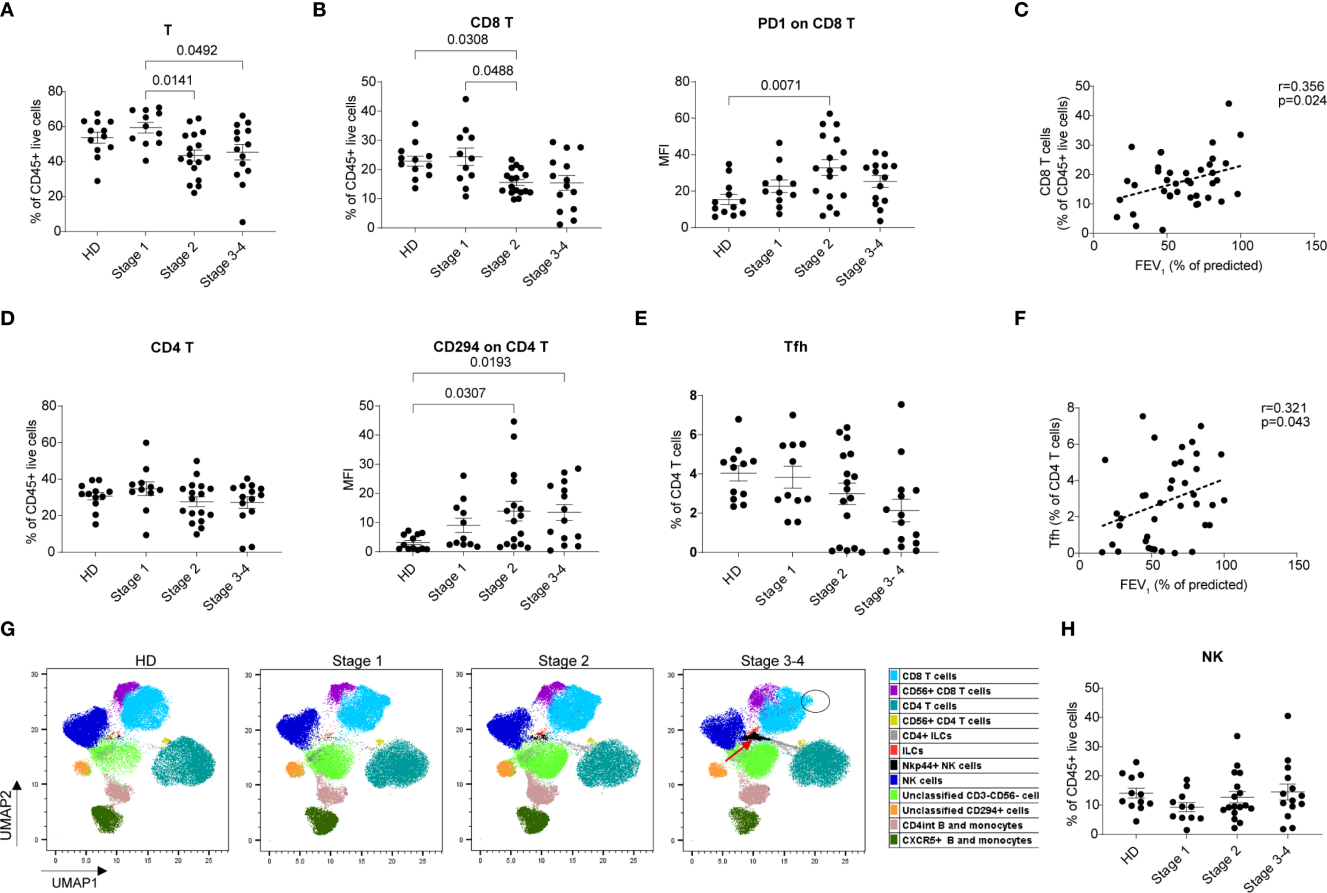
Comparison of NK and T cells in whole blood from different stages of COPD patients and HDs. **(A)** Percentage of total T cells within CD45^+^ live cells. **(B)** Percentage of CD8^+^ T within CD45^+^ live cells (left) and MFI of PD1 on CD8^+^ T cells (right). **(C)** Correlation between CD8^+^ T and FEV_1_. **(D)** Percentage of CD4^+^ T within CD45^+^ live cells (left) and MFI of CD294 on CD4^+^ T cells (right). **(E)** Percentage of Tfh cells within CD4^+^ T cells. **(F)** Correlation between Tfh cells and FEV_1_. **(G)** UMAP analysis of flow panel 2 indicates the alteration of NK cells and T cells with increasing disease severity. UMAP projection of CD45^+^ live cells from 12 HDs and 26 COPD patients (stage 1 n = 8, stage 2 n = 10, stage 3–4 n = 8). **(H)** Percentage of NK cells within CD45^+^ live cells. Scatter dot plots were presented with mean ± SE, the data of 12 HDs and 42 COPD patients (stage 1 n = 11, stage 2 n = 17, stage 3–4 n = 14) were included. Statistical analysis of the comparisons in **(B)** (left), **(D)** (right) and **(H)** were performed using the non-parametric Kruskal-Wallis with Dunn’s multiple comparisons test by GraphPad (version 9.0.0). Statistical analysis of the comparisons in **(A, B)** (right), **(D)** (left) and **(E)** were performed using Ordinary one-way ANOVA with Tukey’s multiple comparisons test by GraphPad (version 9.0.0). *P*-values were denoted and those lower than 0.05 are considered statistically significant. In **(B, F)** Correlation coefficients (R), *P*-value (Pearson’s correlation) were denoted.

We initially observed a substantial reduction in the proportion of total T cells in COPD patients at stage 2 and stage 3–4 relative to stage 1 (**Figure 3A**). Subsequent analyses focusing on CD8^+^ T cells indicated that this decline in total T-cell frequency is likely attributable to a concomitant down-regulation of the CD8^+^ T cell subset (**Figure 3B**). Additionally, we noted an increased expression of PD-1 on CD8^+^ T cells in the patient groups, particularly in stage 2 group compared to HDs, suggesting functional inhibition or exhaustion of CD8^+^ T cells implicated in COPD pathogenesis (**Figure 3B**). Moreover, CD8^+^ T cells percentage inversely correlated with COPD severity, as manifested by FEV_1_ (% of predicted) (**Figure 3C**), highlighting the suppression of the CD8^+^ T cell population as a critical aspect of COPD-related immune alteration.

The percentage of CD4^+^ T cells remained similar between patients and HDs (**Figure 3D**), whereas the CD294 MFI values on CD4^+^ T cells were higher in patient groups, particularly in stage 3–4 group (**Figure 3D**), suggesting the differentiation trajectory toward Th2 cells in the pathogenesis of COPD. The frequency of circulating Tfh cells, which constituted approximately 4% of CD4^+^ T cells in stage1, decreased to ~2% in stages 3–4 (**Figure 3E**). Consistently, it showed positive correlation with FEV_1_ values (**Figure 3F**). The inverse correlation between Tfh cell frequency and COPD severity highlights the importance of Tfh cells in maintaining immune homeostasis during COPD progression.

We then employed UMAP illustration to further confirm the changes observed in CD8^+^ T cells and to analyze the NK cell subsets. The divergence in CD8^+^ T cell distribution between COPD patients and HDs (**Figure 3G**, indicated by the black circle) suggested a potential perturbation in the composition of CD8^+^ T cell subsets in the peripheral blood of COPD patients. Moreover, an additional population of Nkp44^+^ NK cells, which are indicative of activated NK cells (24), was observed in stage 2 and stage 3–4 compared to HDs (**Figure 3G**, indicated by the red arrows). Total NK cell percentage also showed an increasing trend with escalating disease severity (**Figure 3H**). These findings suggest that aberrant activation of certain NK cells could contribute to the acceleration of inflammatory processes and promote disease progression.

**Figure 4:**
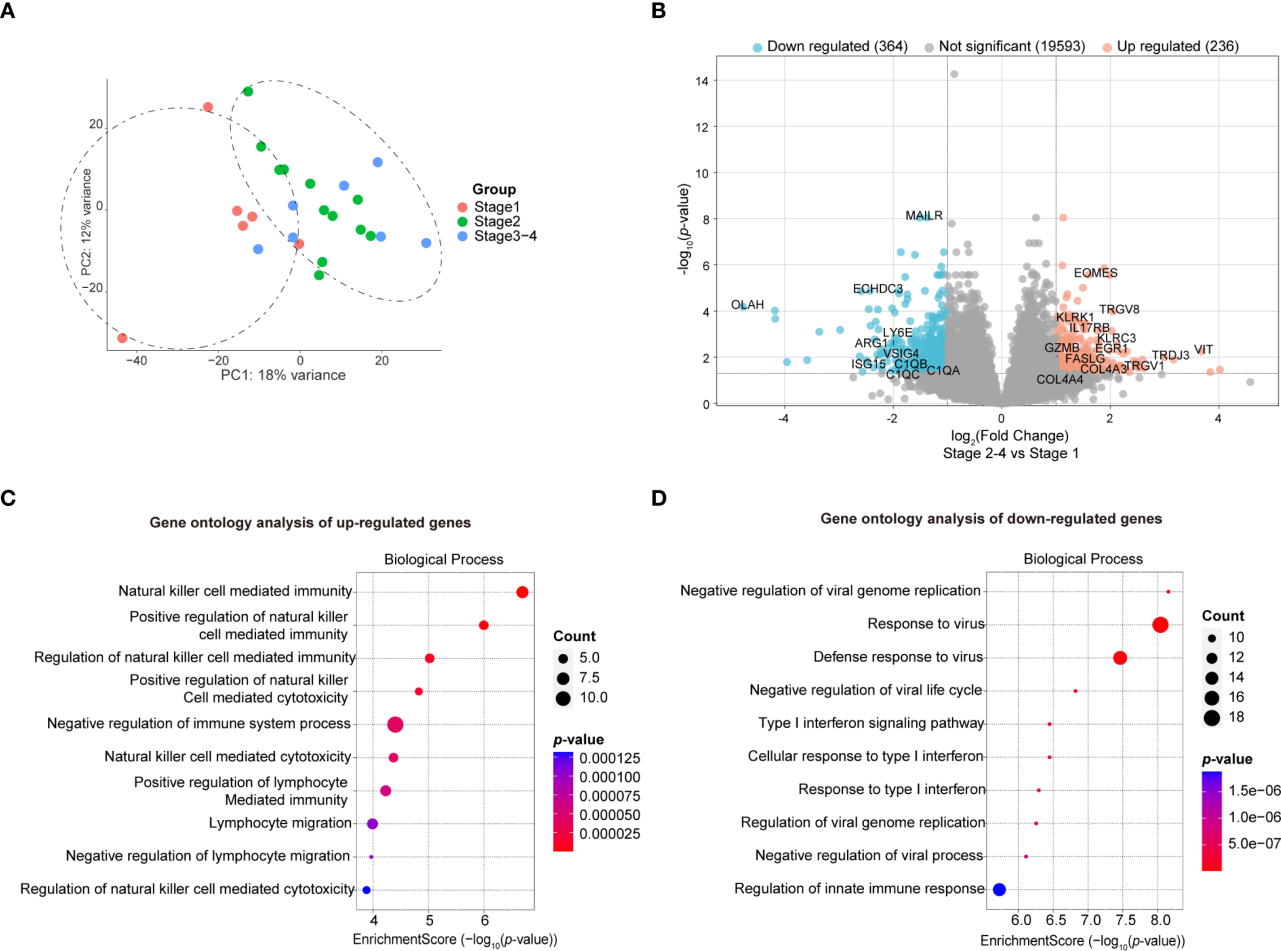
Bulk RNA-seq analysis to compare the transcriptional changes between stage2–4 and stage1 group. **(A)** PCA analysis to show the global variability of gene expression in each individual. (stage 1 n = 6, stage 2 n = 12, stage 3–4 n = 7). **(B)** Volcano plot to show total 600 DEGs between patients of stage2–4 and stage1. (log_2_(FC) < -1 and FDR < 0.05 were indicated by green, and (log_2_(FC) > 1 and FDR < 0.05 were indicated by red). **(C)** Pathway enrichment analysis to show the enriched pathways of the upregulated genes between patients of stage2–4 and stage1 (Enrichment Score > 3). **(D)** Pathway enrichment analysis to show the enriched pathways of the downregulated genes between patients of stage2–4 and stage1 (Enrichment Score > 5.5).

To elucidate the key regulatory factors underlying disease progression, we conducted a targeted analysis of samples among distinct disease groups. PCA revealed a partial segregation of the global transcriptomic signatures between stage 1 and stages 2–4 groups, though with substantial overlap – (**Figure 4A**). Subsequent differential gene expression analysis pinpointed 236 upregulated and 364 downregulated DEGs, (log_2_(FC) > 1 and FDR < 0.05) (**Figure 4B**, **Supplementary Table S4**).

Functional enrichment analysis indicated that immune cells from individuals with advanced disease stages (stages 2-4) exhibited elevated expression of genes predominantly linked to NK cell-mediated immunity (**Figure 4C**, **Supplementary Table S4**). This finding confirms prior observations, revealing aberrant NK cell activation that may induce excessive immune responses, contributing to chronic inflammation and disease progression. Conversely, immune cells from patients with stage 2–4 exhibited downregulation of genes associated with antiviral responses and the type I interferon response (**Figure 4D**, **Supplementary Table S4**), consistent with CD8^+^ T cell suppression. These findings suggest that patients with advanced disease are more susceptible to infections and consequently more likely to suffer exacerbations.

### Imbalance between naïve and memory B cells and the presence of B1 cells in the blood of COPD patients

**Figure 5:**
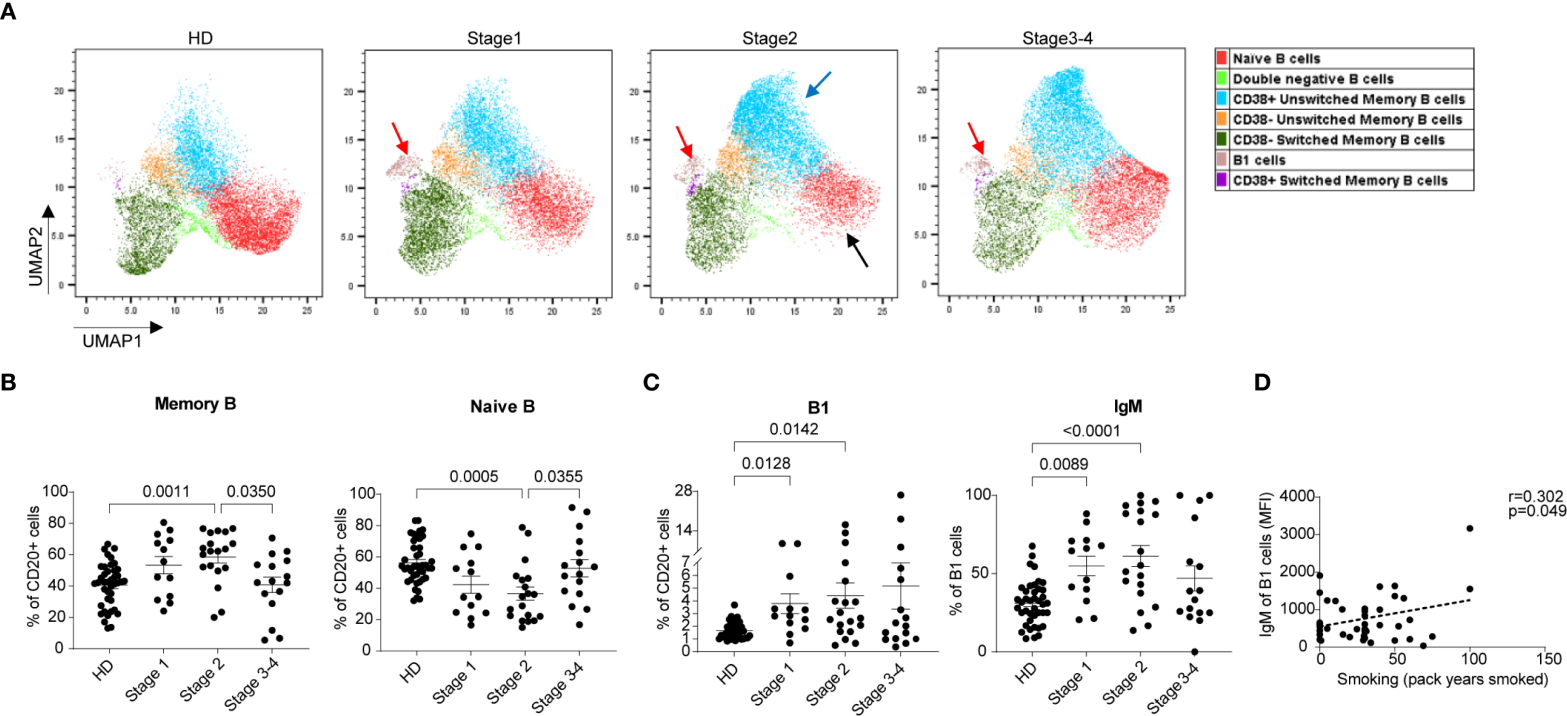
Comparison of B cell subpopulations in whole blood from different stages of COPD patients and HDs. **(A)** UMAP analysis of flow panel 3 reveals the increase of memory B and B1 cells in the blood of COPD patients. UMAP projection of CD45^+^CD3^-^CD20^+^ live cells from 8 HDs and 24 COPD patients (n = 8 for each group). **(B)** Percentage of memory B (left) and naïve B (right) cells within CD20^+^ B cells. **(C)** Percentage of B1 cells and IgM^+^ B1 cells. **(D)** Correlation between IgM expressions on B1 cells and smoking history. Scatter dot plots were presented with mean ± SE, the data of 41 HDs and 48 COPD patients (stage 1 n = 13, stage 2 n = 19, stage 3–4 n = 16) were included. Statistical analysis of the comparisons in **(B)** (left) and **(C)** (left) were performed using the non-parametric Kruskal-Wallis with Dunn’s multiple comparisons test by GraphPad (version 9.0.0). Statistical analysis of the comparisons in **(B)** (right) and **(C)** (right) were performed using Ordinary one-way ANOVA with Tukey’s multiple comparisons test by GraphPad (version 9.0.0). *P*-values were denoted and those lower than 0.05 are considered statistically significant. In **(D)** Correlation coefficients (R), *P*-value (Pearson’s correlation) were denoted.

UMAP analysis of CD45^+^ CD3^-^ CD20^+^ cells delineated the changes within B cell compartments. We firstly identified naïve B cells (CD27^-^ CD43^-^) and memory B cells (CD27^+^ CD43^-^) (**Supplementary Figure S2C**), and observed an apparent increase in the frequency of CD38^+^ unswitched memory B cells and a decrease in the frequency of naïve B cells in all patient groups (**Figure 5A**), with these changes being particularly pronounced in the stage 2 (indicated by blue and black arrows). Consistently, the conventional gating strategy also indicated similar trends in the profiles of total memory and naïve B cells between patients and HDs (**Figure 5B**).

Additionally, a distinct subset of cells, identified as B1 cells (CD20^+^ CD27^+^ CD43^+^) (25), was discerned in the UMAP clustering (indicated by red arrows in **Figure 5A**, **Supplementary Figure S2C**). These B1 cells were relatively infrequent in the peripheral blood of HDs (1.66 ± 0.10% of B cells) but increased in COPD patients (3.79 ± 0.78% in stage 1, 4.41 ± 0.99% in stage 2, and 5.17 ± 1.82% in stage 3-4) (**Figure 5C**). Not only was the proportion of B1 cells increased in COPD patients, but also was IgM expression on B1 cells increased (**Figure 5C**). This increase in IgM expression on B1 cells was further substantiated by a positive correlation with the smoking history of the patients (**Figure 5D**). This correlation underscores the potential role of smoking-induced activation of B1 cells in driving chronic inflammation, thereby contributing to the pathogenesis of COPD.

### Smoking-induced immune perturbations were associated with COPD severity

**Figure 6:**
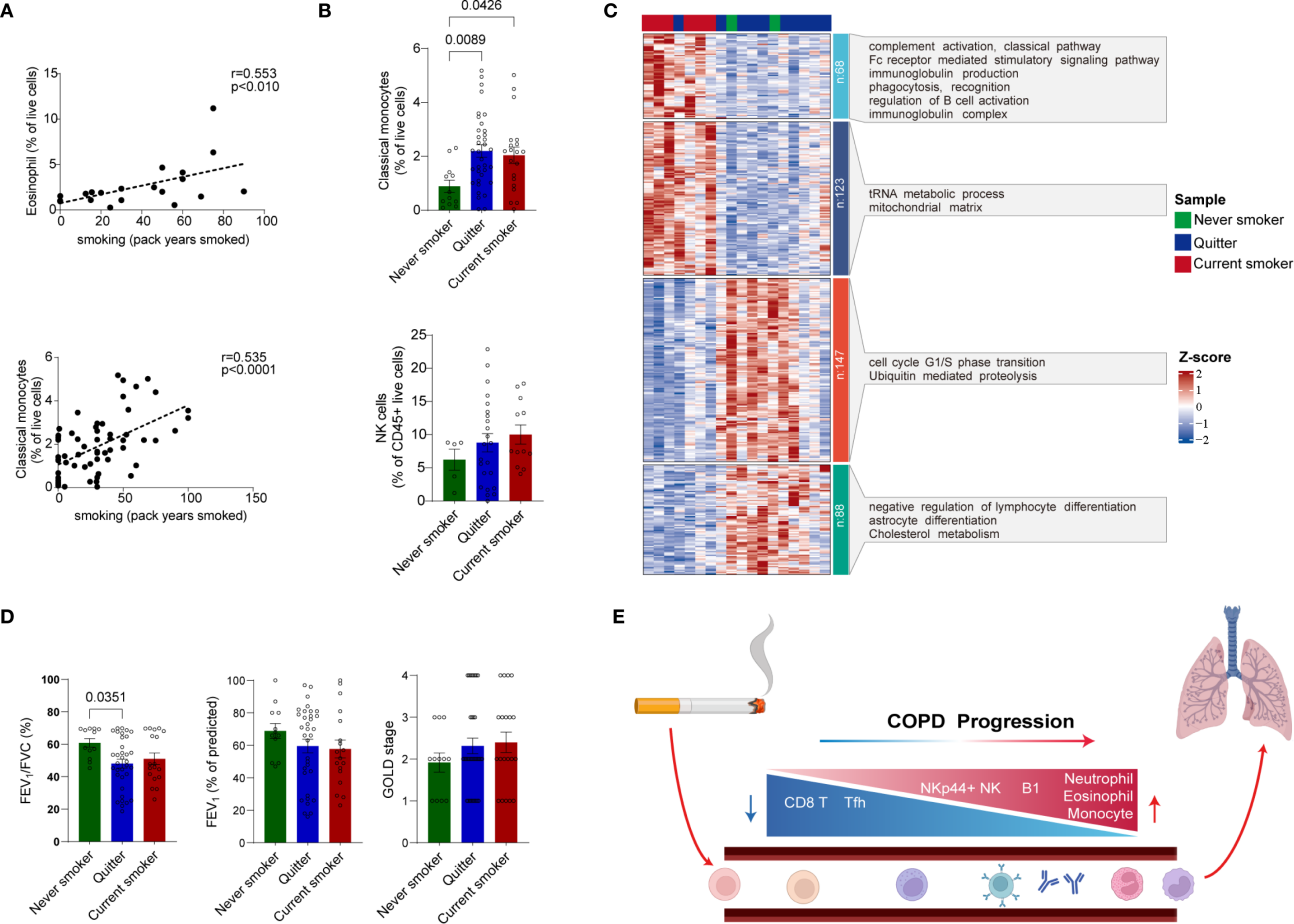
Smoking-induced immune perturbations were associated with COPD severity. **(A)** Correlation between the proportion of eosinophils (upper) and classical monocytes (lower) with smoking history. The data only includes the patients with eosinophil percentage > 1% (mean percentage value of eosinophils among all patients). **(B)** Dot plot showing the comparisons of classical monocytes (upper) and NK cell (lower) proportion among the current smokers, the quitters and the never smokers (for upper panel, current smokers, n = 20; quitters, n = 35; never smokers, n = 12. For lower panel, current smokers, n = 12; quitters, n = 23; never smokers, n = 5). **(C)** Heatmap to show the gene expression of 426 DEGs in current smokers compared to quitters and never smokers (FDR < 0.05) with annotations of enriched pathway and GO terms (current smokers, n = 6; quitters and never smokers, n = 12). **(D)** Dot plot showing the comparison of FEV_1_/FEV (%), FEV_1_ (% of predicted) and GOLD stage among the current smokers, the quitters and the never smokers (current smokers n = 18; quitters, n = 34; never smokers, n = 12). **(E)** Schematic summarizing to show the impact of smoking-induced immune perturbations on COPD progression. Statistical analysis of the comparisons in **(D)** (right) was performed using the non-parametric Kruskal-Wallis with Dunn’s multiple comparisons test by GraphPad (version 9.0.0). Statistical analysis of the comparisons in **(B, D)** (left and middle) were performed using Ordinary one-way ANOVA with Tukey’s multiple comparisons test by GraphPad (version 9.0.0). *P*-values were denoted and those lower than 0.05 are considered statistically significant. In **(A)** Correlation coefficients (R), *P*-value (Pearson’s correlation) were denoted.

In addition to its effects on B cells, smoking exerts a substantial influence on other immune cell subsets. Notably, we observed a significant correlation between the frequency of eosinophils and classical monocytes with smoking history (**Figure 6A**), highlighting the pervasive impact of smoking on immune cell dynamics.

To more precisely elucidate the relationship between smoking-induced immune perturbations and COPD, we meticulously documented the smoking status of patients and classified them into three distinct groups: never smokers, quitters (those with a history of smoking for several years but who had quit for more than one year), and current smokers. Compared with never smokers, both current smokers and quitters exhibited notable increases in the proportion of classical monocytes, as well as increases in NK cell proportions (**Figure 6B**), indicating that smoking-induced perturbations in immune-subset homeostasis persist despite prolonged smoking cessation. Moreover, smoking-induced perturbations of immune-cell homeostasis showed a divergent pattern in stage 2–4 compared with stage 1 (**Supplementary Figure S3**). We propose that relatively preserved immunoregulatory capacity in mild (stage 1) COPD may mitigate smoking-induced immune perturbations, preventing the imbalances seen in moderate-to-severe disease. However, limited sample sizes within strata precluded statistical significance. These findings warrant validation in a larger cohort.

To further elucidate the molecular mechanisms of smoking effects, we captured the transcriptomic changes in stage 2–4 patients with different smoking statuses. Current smokers exhibited distinct transcriptional profiles compared to quitters and never smokers (**Figure 6C**). Gene pathway analysis highlighted substantial upregulation in pathways associated with complement activation, Fc receptor-mediated stimulatory signaling, immunoglobulin production, phagocytosis, and B cell activation in the current smokers (**Figure 6C**, **Supplementary Table S5**). In contrast, genes associated with the cell cycle, ubiquitin-mediated proteolysis, negative regulation of lymphocyte differentiation, astrocyte differentiation, and cholesterol metabolism were more prominently expressed in quitters and never smokers, indicative of a balanced intracorporeal energy metabolism and homeostasis (**Figure 6C**, **Supplementary Table S5**). Although the comparable magnitude of immune-subset perturbation between current smokers and quitters has been shown (**Figure 6B**), transcriptional profiling revealed that the two quitters displayed transcriptomic signatures closely converging with those of never smokers, suggesting that smoking cessation can partially restore systemic immune homeostasis at the transcriptional level (**Figure 6C**).

Ultimately, we compared pulmonary function metrics among the three groups with different smoking statuses. Results revealed that both current smokers and quitters exhibited markedly impaired pulmonary function relative to never smokers, as evidenced by reduced FEV_1_/FVC ratios and lower percentages of predicted FEV_1_ (**Figure 6D**). Consistently, COPD severity grading trended higher in current smokers and quitters than never smokers (**Figure 6D**). Collectively, these findings reiterate the pivotal role of smoking as a key driver of COPD progression.

In summary, we systematically characterized the cellular composition changes associated with smoking status and disease severity, which primarily encompassed the decline of CD8^+^ T cells and Tfh cells, as well as the increase of neutrophils, eosinophils, monocytes, B1 cells, and NKp44^+^ NK cells (**Figure 6E**).

## Discussion

This study provides a comprehensive analysis of systemic immune dysregulation in COPD, integrating high-dimensional flow cytometry and transcriptomic profiling to delineate myeloid-lymphoid imbalances and novel cellular drivers of disease progression. It reveals a pronounced shift toward myeloid cell dominance, marked by elevated neutrophils, eosinophils, and classical monocytes, alongside a decline in lymphoid populations, particularly CD8^+^ T cells and circulating Tfh cells, correlating with disease severity. Notably, we identified aberrant activation of Nkp44^+^ NK cells as a hallmark of severe COPD, implicating innate lymphoid cell hyperactivity in chronic inflammation and tissue damage.

Neutrophils, the most abundant leukocyte cells in the circulation, are a vital component of innate immunity. A previous study assessed a panel of activation and maturation markers, namely CD11b, CD35, and CD11c, on circulating neutrophils in patients with COPD and showed no significant differences relative to healthy controls (26). However, an independent study observed elevated CD11b expression on sputum neutrophils in smokers with COPD (27). In the present study, we documented a pronounced increase in the percentage of CD11c^+^ neutrophils in individuals with mild-to-moderate COPD compared to healthy controls. These disparate findings underscore the phenotypic heterogeneity of neutrophils across distinct COPD cohorts. CD11c is widely recognized as a marker of neutrophil maturation and, in addition, has been shown to independently modulate reactive oxygen species (ROS) generation (28). Future investigations should therefore incorporate a broader array of activation and oxidative burst indicators, such as CD62L, CD11b, and CD54, to elucidate more comprehensively the contribution of neutrophil functional states to COPD pathobiology and disease progression.

COPD is typically associated with neutrophilic airway inflammation, yet a significant subset of patients (32%-40%) exhibit eosinophilic inflammation, as indicated by increased eosinophils in blood or sputum (12, 29, 30). In this study, elevated blood eosinophils in COPD patients demonstrate the prevalence of eosinophilic inflammation in the patient population. Moreover, a positive correlation between smoking history and eosinophil levels in some patients was revealed, suggesting that smoking may be a primary driver of eosinophilic inflammation. Recent evidence increasingly supports the idea that peripheral blood eosinophils may predict the response to inhaled corticosteroids for the prevention of exacerbations (13). Therefore, understanding the eosinophil associated pathogenetic pathways in COPD would be valuable in providing useful insights into effective treatments for these patients.

COPD is also often characterized by an accumulation of macrophages in airways and lungs (31–33). Previous studies have shown that the proportion of intermediate monocytes in the blood is elevated in COPD patients, and hypothesized that the intermediate monocytes differentiate into M2 macrophages in the lung (8), potentially exacerbating tissue remodeling and contributing to tissue damage in COPD. In this study, significant increases in both intermediate and classical monocyte populations were observed. Classical monocytes can differentiate into macrophages in inflamed lung tissue (34), which may partially account for the higher lung macrophage levels in COPD. Additionally, the expression of HLA-DR on classical monocytes decreased significantly with increasing disease severity, suggesting a possible functional impairment or immunosuppression of these monocytes (35). Intriguingly, classical monocytes alone displayed a significantly positive correlation with the smoking history in COPD patients. This suggests that the circulating classical monocyte population may be more responsive to cigarette exposure than intermediate or non-classical monocytes.

Beyond myeloid cell changes, lymphocytes also contribute to the inflammatory response in COPD (36–38). While the increased number of CD8^+^ T cells in the lung correlates with airflow obstruction in COPD (39), the studies worked with peripheral blood have yielded inconsistent results, with most reporting no significant difference in CD8^+^ T cell quantity between COPD patients and healthy controls (39). Only a few studies reported changes in the number of peripheral CD8^+^ T cells in COPD patients (10, 11). Discrepancies may arise from varying detection methods and control subject characteristics. Here, we employed multicolor flow cytometry and transcriptional profiling to elucidate the impact of reduced CD8^+^ T cell numbers and functional impairment on COPD progression.

NK cells, cytotoxic lymphocytes linked to COPD pathogenesis (40, 41), expand in the pulmonary compartment during disease progression (24). However, their role in systemic circulation remains ambiguous (41). While one study has shown no difference in NK cell frequency in peripheral blood between COPD patients and healthy controls (42), others observed reduced NK cell frequency in patients (5). Our study identified Nkp44^+^ NK cells (negligible in the healthy controls) in severe COPD cases, suggesting activated NK cells drive disease progression. Transcriptional profiling analysis also revealed significant enrichment of genes associated with NK cell activation. Previous studies emphasized the importance of NK cell-activating receptors in driving pulmonary inflammation and emphysema in COPD (41, 43). For instance, Finch et al. reported increased CD69^+^ NK cells in the lung of a murine COPD model (44). Furthermore, smoking exposure has been shown to enhance NK cell priming and activation (45, 46). Synthesizing these insights, our work proposes that smoking-induced chronic inflammation disrupts immune homeostasis, creating an inflammatory imbalance that drives NK cell activation and maturation.

Our study has some limitations. For example, the inability to provide a specific cell count per microliter of blood means that our results are presented as percentages of the parent population, complicating direct comparisons with other studies. Another limitation is the lack of functional testing of the defined cell subsets, making it difficult to determine whether the observed perturbation in cell numbers of specific subsets is a consequence of systemic inflammation in COPD or facilitates disease progression.

Collectively, our study demonstrated that various immune cell populations from both myeloid and lymphocyte lineages in the blood are associated with COPD progression. Aberrant activation of NK cells is the predominant factor contributing to disease progression. This finding underscores the critical role of NK cell dysregulation in the pathogenesis of the disease and highlights the potential for targeted interventions aimed at modulating NK cell activity to mitigate disease severity.

## Data Availability

The original contributions presented in the study are included in the article/**Supplementary Material**. Original data or other related materials are available on request from the corresponding author, Ling Ni (lingni@mail.tsinghua.edu.cn). The data presented in the study are deposited in NCBI's Gene Expression Omnibus repository, accession number GSE306950 (https://www.ncbi.nlm.nih.gov/geo/query/acc.cgi?acc=GSE306950).
