# Supplementary material for: Dysregulated NK cell activation and myeloid-lymphoid imbalance underpin COPD progression: insights from high-dimensional immune profiling and smoking-induced immune remodeling
Source: Front Immunol. 2025 Sep 19;16:1623319. doi: 10.3389/fimmu.2025.1623319 (PMC12491182; doi:10.3389/fimmu.2025.1623319)
Supplement: Supplementary file 6 [file DataSheet6.pdf]

Supplementary Material

Supplementary Figures:

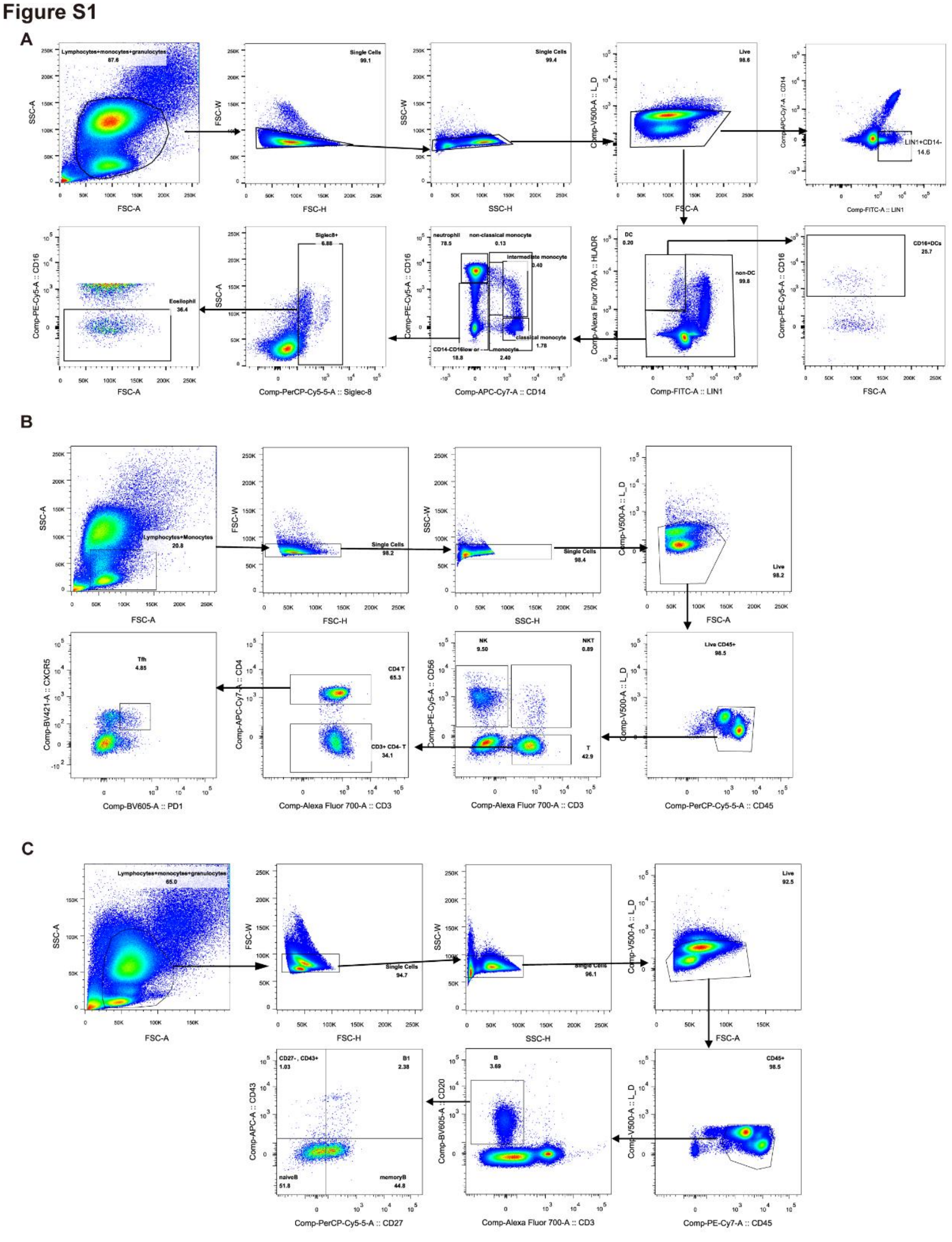

Figure S1. Representative flow cytometry plots of the gating strategy for Panel 1 (A), panel 2 (B) and panel 3 (C).

Figure S2

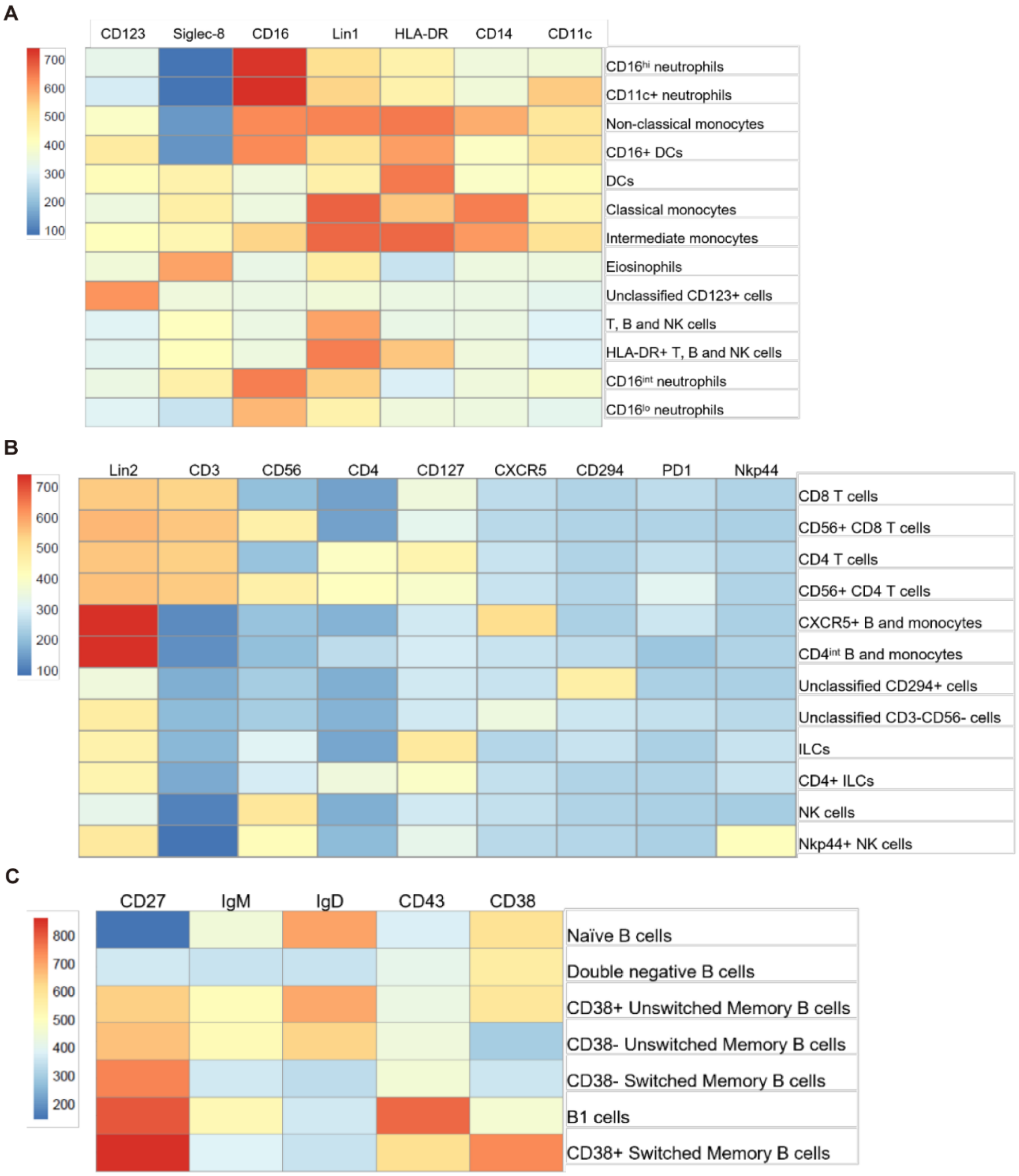

Figure S2. Heatmap representation of mean fluorescence intensity of each marker across cell subsets clustered by FlowSOM. (A) for Panel 1, (B) for Panel 2, (C) for Panel 3.

Figure S3

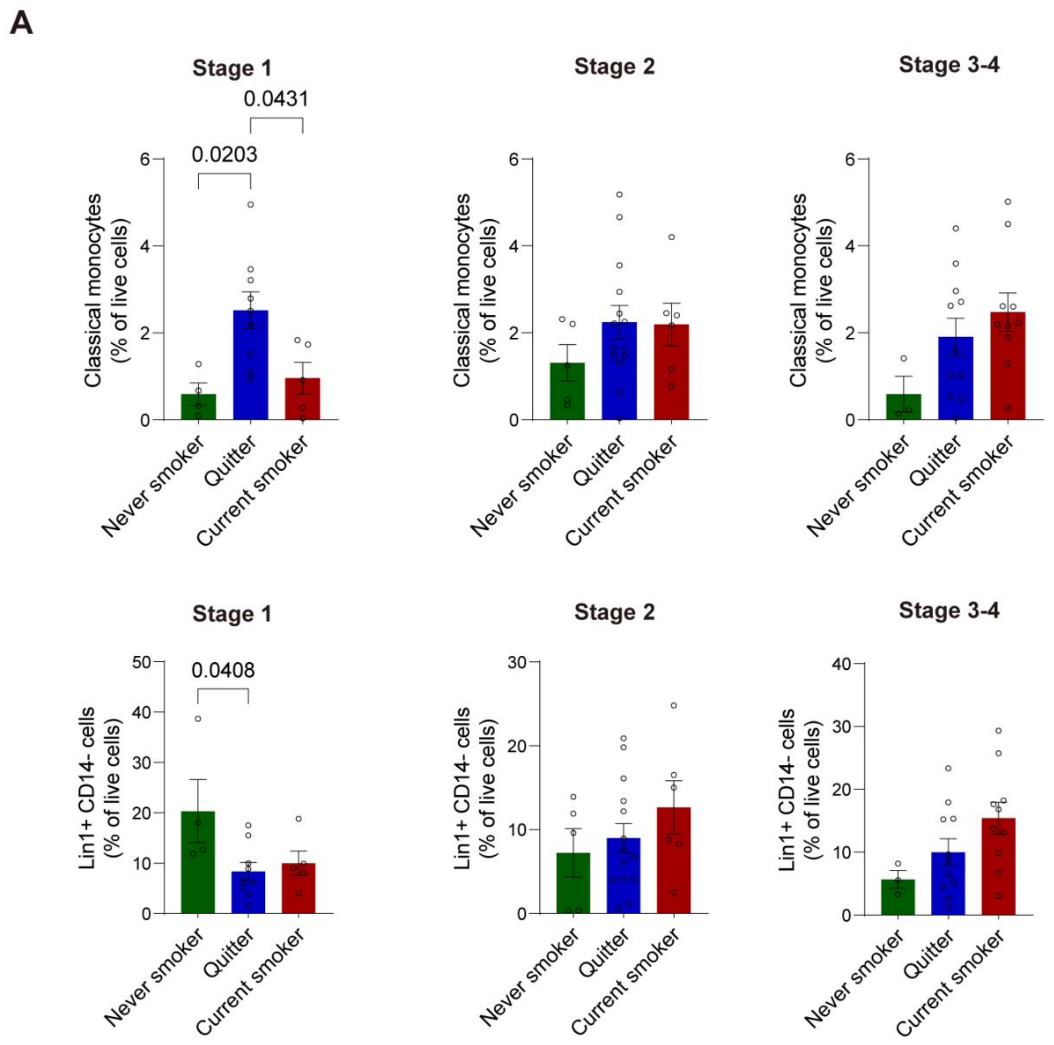

Figure S3. Bar charts to show the proportion changes of classical monocytes ($CD14^{+}CD16^{-}$) and total lymphocytes ($Lin1^{+}CD14^{-}$) cells across smoking status categories within each COPD severity stage.
